# Association between anion gap trajectories and mortality in patients with acute cholangitis: a cohort study

**DOI:** 10.1038/s41598-026-48549-5

**Published:** 2026-04-10

**Authors:** Yanhua Chen, Guirong Xiao, Ling Mou

**Affiliations:** 1 Department of Pharmacy, Hospital of Chengdu University of Traditional Chinese Medicine, Chengdu, China; 2 School of Clinical Medicine, Chengdu University of Traditional Chinese Medicine, Chengdu, China; 3 Department of Pharmacy, West China Hospital, Sichuan University, Chengdu, China; 4 Department of Pharmacy, Traditional Chinese Medicine Hospital of Meishan, Meishan, China

**Keywords:** Acute cholangitis, Anion gap, Trajectory, Critically ill patients, All-cause mortality, Diseases, Gastroenterology, Medical research, Risk factors

## Abstract

Prior studies have suggested that baseline anion gap (AG) is associated with prognosis in acute cholangitis (AC). however, the association between AG trajectories after ICU admission and clinical outcomes remains unclear. Using data from the Medical Information Mart for Intensive Care IV (MIMIC-IV), we included 861 ICU-admitted patients with AC. AG measurements during the first 96 h after ICU admission were extracted. Latent class growth modeling (LCGM) identified four AG trajectory classes: Class 1 (persistently low), Class 2 (persistently moderate), Class 3 (persistently elevated), and Class 4 (persistently elevated despite decline). Kaplan–Meier analysis and multivariable Cox regression after comprehensive adjustment for confounders were used to assess associations with 28-day and 90-day all-cause mortality. Over all 28-day and 90-day mortality were 16.7% and 26.5%, respectively. Compared with Class 1, Class 3 and Class 4 showed higher adjusted mortality risks (28-day: Class 3 h 2.79, 95% CI 1.55–5.00, *p* = 0.001; Class 4 h 4.76, 95% CI 2.33–9.72, *p* < 0.001; 90-day: Class 3 h 2.34, 95% CI 1.49–3.68, *p* < 0.001; Class 4 h 4.12, 95% CI 2.31–7.36, *p* < 0.001). These findings suggest that dynamic AG monitoring during the early ICU course may help identify high-risk AC patients and inform clinical management.

**Supplementary Information:**

The online version contains supplementary material available at 10.1038/s41598-026-48549-5.

## Introduction

Acute cholangitis (AC) is a life-threatening hepatobiliary emergency caused by bacterial infection secondary to biliary obstruction, most commonly due to biliary stones, strictures, or malignancies^1,2^. Increased biliary pressure compromises the bile duct-venous barrier, facilitating bacterial translocation and endotoxin release into the systemic circulation, thereby triggering a profound inflammatory response. The classic Charcot’s triad (fever, jaundice, right upper quadrant pain) manifests in 50–70% of cases. Critically ill patients may rapidly progress to Reynolds’ pentad, characterized by altered mental status and septic shock^3–5^. The Tokyo Guidelines (TG18) emphasize early biliary drainage and appropriate antimicrobial therapy as cornerstone strategies for improving AC outcomes^6^. Current prognostic assessment predominantly relies on inflammatory markers such as C-reactive protein (CRP) and procalcitonin (PCT)^7,8^. However, these biomarkers exhibit some limitations, including low specificity, susceptibility to confounding factors, and particularly limited predictive value after biliary decompression. Consequently, an accessible, cost-effective laboratory indicator enabling rapid risk stratification and individualized therapeutic decision-making is urgently needed.

Serum anion gap (AG) represents a fundamental biomarker for evaluating acid-base homeostasis^9,10^. AG elevation frequently accompanies critical conditions including lactic acidosis and ketoacidosis, reflecting the severity of systemic hypoperfusion, tissue hypoxia, and metabolic dysregulation^11–13^. Recent evidence has established its role as an independent prognostic predictor in critically ill patients^14^. This association has been substantiated across diverse conditions, including sepsis, pancreatitis, and acute pulmonary edema^15–17^. The study by Huang et al. demonstrated that admission AG is independently associated with 28-day mortality in AC patients, establishing its disease-specific prognostic value^18^. However, baseline AG may be affected by sampling time, pre‑hospital interventions, or transient metabolic disturbances, and therefore may not fully capture the evolving disease process or response to therapy. In contrast, serial AG monitoring and trajectory analysis can reveal temporal patterns, such as persistent elevation, delayed normalization, or rapid recovery, thereby helping to distinguish patient subgroups with different treatment responses and prognose.

This retrospective cohort study utilizes the Medical Information Mart for Intensive Care IV (MIMIC-IV) database to characterize AG trajectories as prognostic indicators of 28-day and 90-day mortality in critically ill AC patients. We hypothesize that AG trajectory is associated with mortality and that modeling the dynamic changes of AG can help improve risk stratification and inform evidence-based therapeutic decision-making.

## Methods

### Data source

The study analyzed data from the MIMIC-IV database (version 3.1), which comprises de-identified health records of intensive care unit (ICU) patients at Beth Israel Deaconess Medical Center from 2008 to 2022^19^. The repository included 94,458 ICU admissions representing 65,366 unique individuals. Ethics oversight committees at the Massachusetts Institute of Technology and Beth Israel Deaconess Medical Center granted approval for database utilization and waived informed consent requirements due to thorough anonymization. One author (Y.C.) obtained access after completing the CITI Program training (certification ID: 70325517). This investigation adhered to the ethical guidelines outlined in the Declaration of Helsinki.

### Study population

**Fig. 1:**
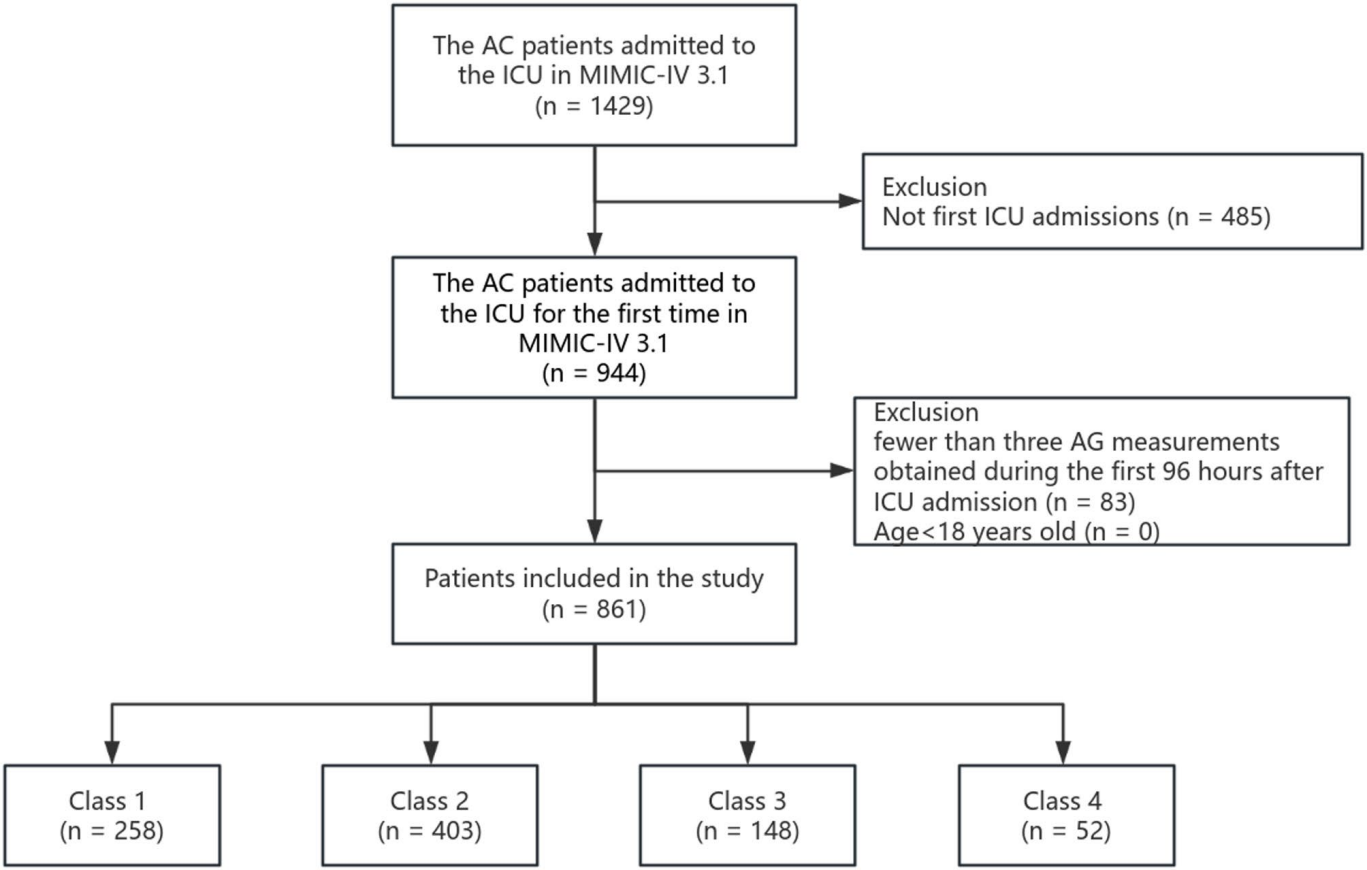
Study flowchart.

Patients with AC were identified within the MIMIC-IV database utilizing International Classification of Diseases (ICD) diagnostic codes: ICD-9 (576.1) and ICD-10 (K83.0, K83.09, K80.3, K80.30, K80.31, K80.32, K80.33). Exclusion criteria included: (1) non-first admission to the ICU, (2) age < 18 years, and (3) fewer than three AG measurements obtained during the first 96 h after ICU admission. After these exclusions, the final cohort comprised 861 patients with AC. The complete patient selection workflow is depicted in Fig. 1.

### Data extraction

Structured Query Language (SQL) was employed to extract relevant clinical variables for the cohort. These included demographic characteristics (age, sex, race), vital signs (heart rate [HR], respiratory rate [RR], temperature, oxygen saturation [SpO_2_]), laboratory parameters (hemoglobin, platelet count [PLT], white blood cell [WBC], bicarbonate, blood urea nitrogen [BUN], chloride, creatinine, sodium, international normalized ratio [INR], alanine aminotransferase [ALT], aspartate aminotransferase [AST], total bilirubin), etiology of AC (biliary stones, biliary malignancy), clinical interventions and complications (acute kidney injury [AKI], continuous renal replacement therapy [CRRT], procedures of biliary drainage), comorbid conditions (hypertension, congestive heart failure [CHF], renal disease, chronic obstructive pulmonary disease [COPD], diabetes, and sepsis conforming to Sepsis-3 criteria), and illness severity indices (Charlson Comorbidity Index [CCI], Simplified Acute Physiology Score II [SAPS II], Sequential Organ Failure Assessment [SOFA]).

### Research variable and outcomes

AG constituted the primary exposure variable in this investigation. AG trajectories were characterized using serial measurements within 96 h of ICU admission. Distinct trajectory classes were identified via latent class growth modeling (LCGM). The primary endpoints were 28-day and 90-day all-cause mortality among AC patients, which were ascertained from mortality records and follow-up data in the MIMIC-IV database.

### Covariate assessment

The inclusion of covariates was primarily based on evidence from previous studies and clinical experience. To enhance the objectivity of selection, two statistical criteria were additionally applied: first, the change-in-estimate method, where a variables was retained if its inclusion resulted in a ≥ 10% change in the estimated risk ratio; and second, univariate screening, whereby variables significantly associated with the outcome (*p* < 0.1) in univariate analysis were included in the candidate set^20^. The final covariates were determined through comprehensive evaluation based on the above criteria.

### Statistical analysis

Statistical analyses employed descriptive methods to evaluate baseline characteristics across AG trajectory groups. Continuous variables underwent normality assessment using histograms, Q-Q plots, and Kolmogorov-Smirnov tests. Normally distributed data were presented as mean ± standard deviation (SD), while skewed variables were reported as medians with inter quartile ranges. Group comparisons for continuous variables were performed using one‑way analysis of variance (ANOVA) or the Kruskal-Wallis test based on distribution characteristics. Categorical data were expressed as frequencies (percentages), and analyzed via chi-square test or Fisher’s exact tests where appropriate. Kaplan-Meier curves were plotted to illustrate survival differences across AG trajectory groups, with between-group comparisons assessed using log-rank tests. Cox proportional hazards regression quantified associations between distinct AG trajectories and mortality risk in AC patients, calculating hazard ratios (HR) with 95% confidence intervals (95% CI) for 28-day and 90-day all-cause mortality. Four sequential regression models were constructed: Model I (unadjusted); Model II (adjusted for age, race, and sex); Model III (adjusted for Model II covariates plus etiology of AC, AKI, renal disease, procedures of biliary drainage, hypertension, diabetes, and sepsis); and Model IV (adjusted for Model III covariates plus bilirubin, hemoglobin, WBC, ALT, AST, CCI, SAPS II and SOFA).

Subgroup analyses evaluated AG trajectory-mortality associations across defined strata: age (< 65 vs. ≥ 65 years), sex, diabetes, and hypertension. All statistical tests were two-sided, with *p* < 0.05 considered statistically significant. Analyses were performed using R 4.2.2 (https://www.r-project.org, R Foundation) and Free Statistics software (version 2.2)^21–23^.

## Results

### Trajectory model selection

**Fig. 2:**
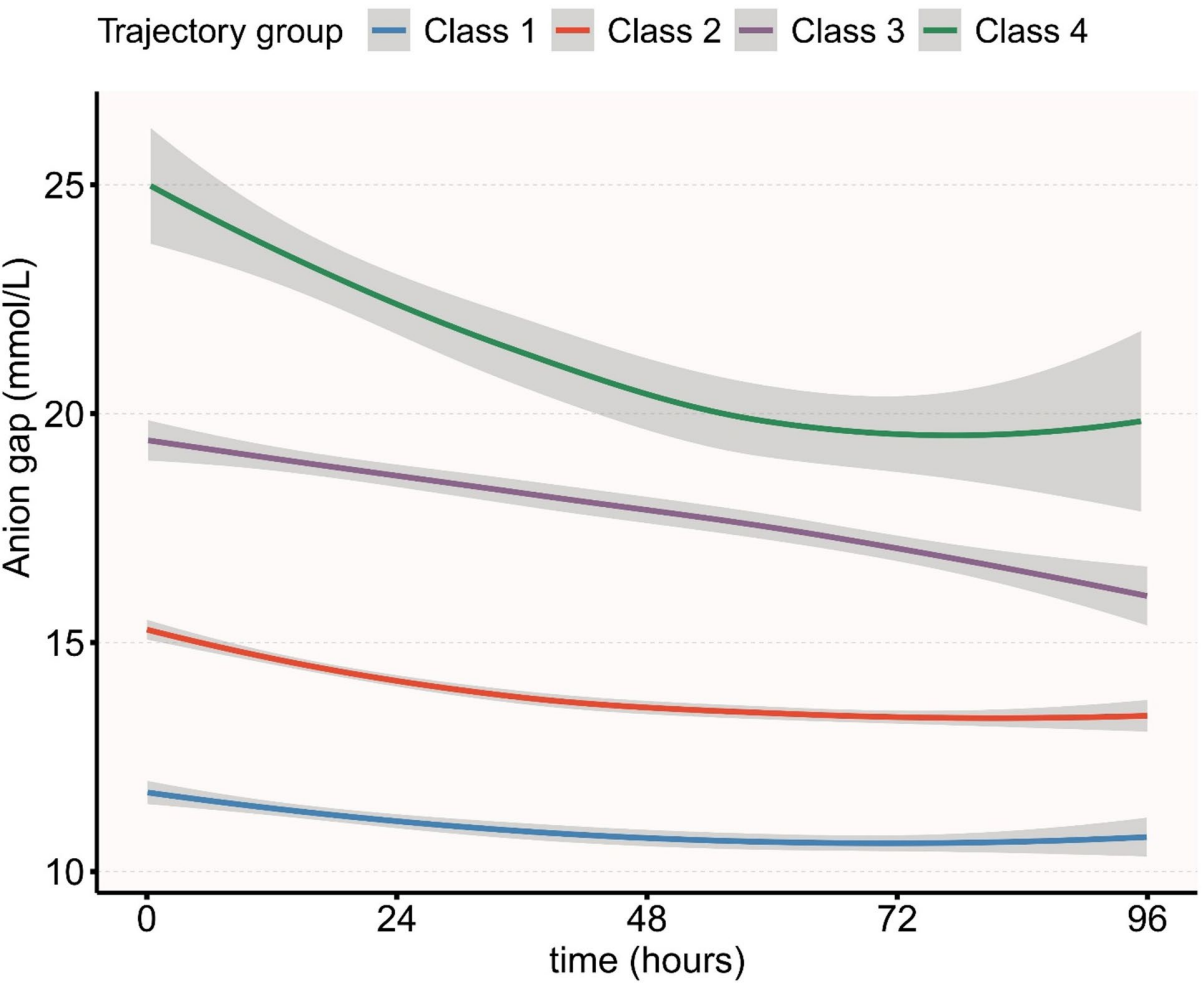
Anion gap trajectory during the first 96 h after ICU admission.

Optimal trajectory classification was determined by evaluating 2- to 5-class models using Akaike Information Criterion (AIC), Bayesian Information Criterion (BIC), sample size-adjusted BIC (SABIC), and entropy (Supplementary Table S1). The four-trajectory model demonstrated superior fit compared to 2- or 3-class alternatives, meeting adequacy criteria of > 5% cohort representation and posterior probability > 0.7 for each trajectory (Supplementary Table S2). LCGM of serial AG measurements identified four distinct trajectories: Class 1 (persistently low), Class 2 (persistently moderate), Class 3 (persistently elevated), and Class 4 (persistently elevated despite decline) (Fig. 2).

### Patient baseline characteristics

Table 1 summarizes the baseline profiles of 861 AC patients grouped by AG trajectory patterns. The cohort had a mean age of 72.2 ± 14.6 years, with 53.0% (*n* = 456) males and 84.2% (*n* = 725) meeting sepsis criteria. Overall, 79.1% (*n* = 681) of patients received biliary drainage, including endoscopic retrograde cholangiopancreatography (ERCP), percutaneous transhepatic biliary drainage (PTBD), or surgical drainage. Compared to Classes 1 and 2, Classes 3 and 4 exhibited elevated levels of HR, RR, WBC, BUN, creatinine, AST, total bilirubin, and SOFA scores. Concurrently, AKI incidence progressively increased, peaking at 92.3% in Class 4. Mortality exhibited stepwise escalation across groups: 28-day all-cause mortality rates were 8.9% (Class 1), 10.2% (Class 2), 31.8% (Class 3), and 63.5% (Class 4); 90-day mortality was 19.4% in Classes 1–2 versus 41.9% (Class 3) and 73.1% (Class 4).

**Table 1:** Baseline characteristics of patients by AG trajectory group.

Characteristics	Total (*n* = 861)	Class 1 (*n* = 258)	Class 2 (*n* = 403)	Class 3 (*n* = 148)	Class 4 (*n* = 52)	*P*-value
Demographics						
Age (years)	72.2 ± 14.6	70.5 ± 13.9	73.4 ± 15.1	72.2 ± 14.3	70.8 ± 14.6	0.083
Sex, male n (%)	456 (53.0)	133 (51.6)	199 (49.4)	94 (63.5)	30 (57.7)	0.025
Race, white n (%)	604 (70.2)	187 (72.5)	283 (70.2)	99 (66.9)	35 (67.3)	0.655
Vital signs						
HR (bpm)	86.1 ± 16.5	83.3 ± 16.3	85.6 ± 15.8	89.5 ± 17.6	94.1 ± 15.9	< 0.001
RR (bpm)	19.8 ± 3.8	18.9 ± 3.4	19.9 ± 3.6	20.3 ± 4.3	22.0 ± 3.7	< 0.001
Temperature (°C)	37.4 ± 0.7	37.4 ± 0.8	37.4 ± 0.7	37.4 ± 0.8	37.4 ± 0.8	0.722
SpO_2_ (%)	96.3 ± 2.1	96.7 ± 1.7	96.3 ± 1.7	96.2 ± 2.4	95.4 ± 4.2	< 0.001
Laboratory tests						
Hemoglobin (g/dL)	10.0 ± 2.1	9.6 ± 2.0	10.2 ± 2.0	10.1 ± 2.1	9.3 ± 2.3	< 0.001
PLT(10³/µL)	175.1 ± 102.9	177.8 ± 108.0	170.2 ± 87.0	185.4 ± 118.5	170.0 ± 137.5	0.437
WBC (10³/µL)	17.4 ± 10.3	14.7 ± 7.8	17.3 ± 8.9	21.1 ± 15.4	21.2 ± 9.7	< 0.001
Bicarbonate (mmol/L)	20.1 ± 4.6	22.2 ± 3.8	20.6 ± 3.7	17.1 ± 4.4	13.6 ± 4.8	< 0.001
BUN (mg/dL)	23.5 (16.0, 39.0)	17.0 (13.0, 25.0)	22.0 (16.0, 33.0)	46.0 (31.0, 65.2)	50.0 (37.8, 85.2)	< 0.001
Chloride (mmol/L)	102.0 ± 6.3	102.9 ± 6.3	102.5 ± 5.6	99.9 ± 7.3	99.0 ± 7.1	< 0.001
Creatinine (mg/dL)	1.2 (0.8, 1.8)	0.9 (0.7, 1.2)	1.2 (0.9, 1.6)	2.3 (1.6, 3.9)	2.5 (1.5, 5.0)	< 0.001
Sodium (mmol/L)	136.4 ± 5.0	136.3 ± 4.9	136.9 ± 4.5	135.2 ± 5.9	135.7 ± 5.9	0.004
INR	1.8 ± 1.2	1.6 ± 0.7	1.7 ± 1.1	2.3 ± 1.8	2.4 ± 1.6	< 0.001
ALT (IU/L)	129.0 (59.0, 256.0)	85.0 (38.0, 155.0)	156.0 (75.0, 287.2)	132.5 (62.8, 284.2)	218.5 (112.0, 555.8)	< 0.001
AST (IU/L)	141.0 (72.0, 286.0)	99.0 (52.0, 174.0)	156.5 (80.0, 293.5)	180.0 (79.8, 350.2)	316.0 (196.0, 1126.2)	< 0.001
Total bilirubin (mg/dL)	4.1 (2.2, 6.9)	3.2 (1.4, 5.5)	4.1 (2.4, 6.7)	5.2 (2.9, 8.9)	6.2 (3.4, 14.7)	< 0.001
Etiology of acute cholangitis						0.005
Biliary stones, n (%)	400 (46.5)	98 (38)	214 (53.1)	69 (46.6)	19 (36.5)	
Biliary malignancy, n (%)	161 (18.7)	58 (22.5)	57 (14.1)	30 (20.3)	16 (30.8)	
Both biliary stones and malignancy, n (%)	21 (2.4)	8 (3.1)	9 (2.2)	4 (2.7)	0 (0.0)	
Other	279 (32.4)	94 (36.4)	123 (30.5)	45 (30.4)	17 (32.7)	
Clinical course						
AKI, n (%)	602 (69.9)	154 (59.7)	274 (68)	126 (85.1)	48 (92.3)	< 0.001
CRRT, n (%)	15 (1.7)	0 (0)	1 (0.2)	8 (5.4)	6 (11.5)	< 0.001
Procedures of biliary drainage, n (%)	681 (79.1)	190 (73.6)	336 (83.4)	119 (80.4)	36 (69.2)	0.006
Comorbidities						
Hypertension, n (%)	359 (41.7)	79 (30.6)	170 (42.2)	87 (58.8)	23 (44.2)	< 0.001
CHF, n (%)	209 (24.3)	42 (16.3)	100 (24.8)	53 (35.8)	14 (26.9)	< 0.001
Renal disease, n (%)	184 (21.4)	27 (10.5)	83 (20.6)	64 (43.2)	10 (19.2)	< 0.001
COPD, n (%)	213 (24.7)	61 (23.6)	99 (24.6)	42 (28.4)	11 (21.2)	0.663
Diabetes, n (%)	300 (34.8)	82 (31.8)	137 (34)	60 (40.5)	21 (40.4)	0.260
Sepsis, n (%)	725 (84.2)	209 (81)	330 (81.9)	136 (91.9)	50 (96.2)	0.001
Severity scores						
CCI	6.7 ± 3.0	6.4 ± 3.0	6.4 ± 2.9	7.7 ± 3.1	7.6 ± 2.7	< 0.001
SAPS II	43.0 ± 14.3	37.8 ± 11.7	40.0 ± 11.6	53.2 ± 13.9	63.5 ± 14.7	< 0.001
SOFA	6.6 ± 3.5	5.3 ± 2.9	6.1 ± 2.9	8.9 ± 3.5	11.1 ± 4.4	< 0.001
Outcome						
28-day mortality, n (%)	144 (16.7)	23 (8.9)	41 (10.2)	47 (31.8)	33 (63.5)	< 0.001
90-day mortality, n (%)	228 (26.5)	50 (19.4)	78 (19.4)	62 (41.9)	38 (73.1)	< 0.001

HR, heart rate; Bpm, beats per minute; RR, respiratory rate; SpO_2_, peripheral oxygen saturation; PLT, platelet; WBC, white blood cell; BUN, blood urea nitrogen; INR, international normalized ratio; ALT, alanine aminotransferase; AST, aspartate aminotransferase; AKI, acute kidney injury; CRRT, continuous renal replacement therapy; CHF, congestive heart failure; COPD, chronic obstructive pulmonary disease; CCI, Charlson Comorbidity Index; SAPS II, Simplified Acute Physiology Score II; SOFA, Sequential Organ Failure Assessment.

### Association between AG trajectories and mortality

**Fig. 3:**
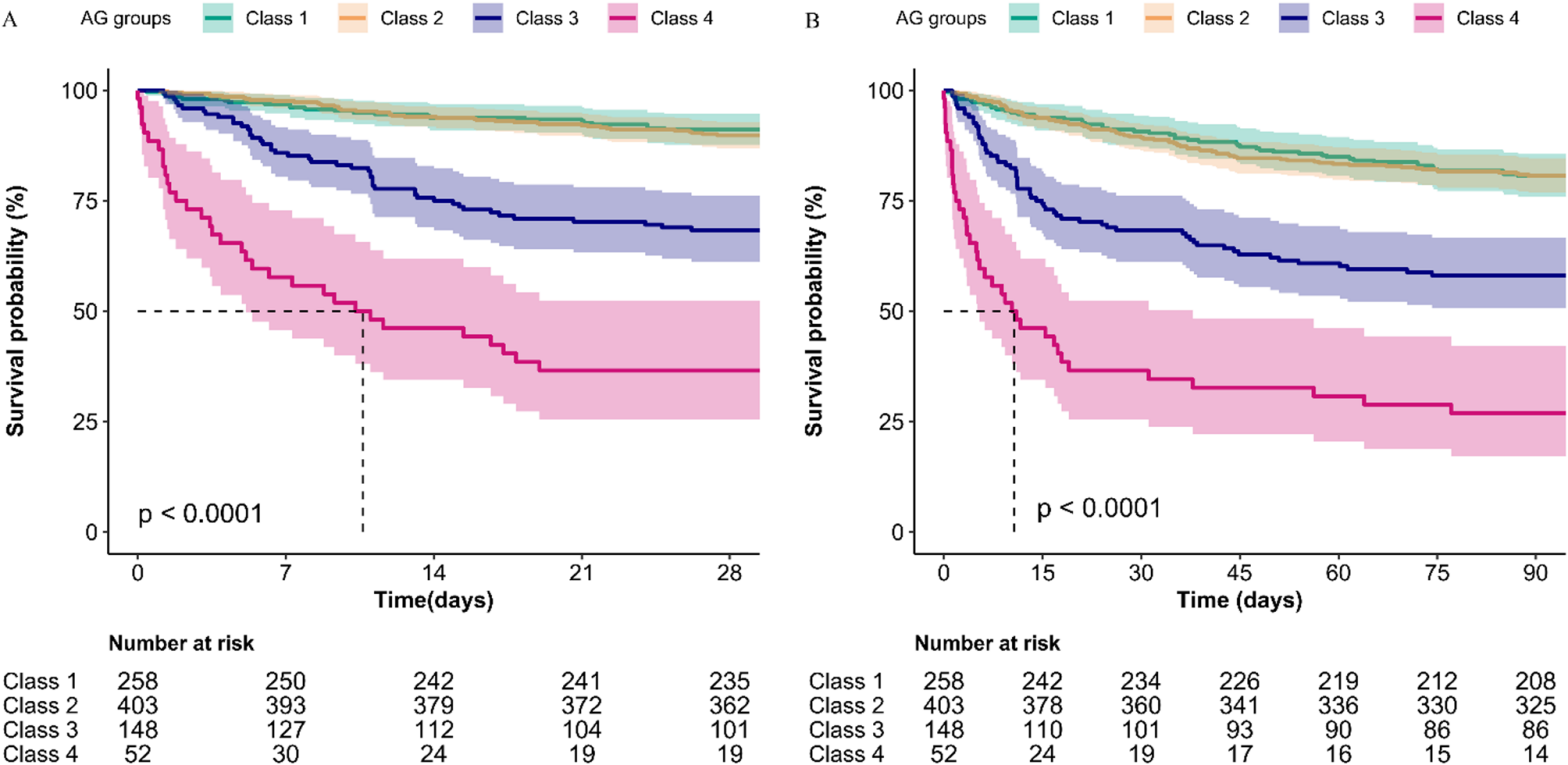
Kaplan-Meier survival curves stratified by anion gap trajectory classes: (**A**) 28-day (**B**) 90-day.

Kaplan-Meier survival curves (Fig. 3) demonstrated significant survival disparities across AG trajectory groups (log-rank test, *p* < 0.0001). Class 4 exhibited the highest mortality at both 28 and 90 days, followed by Class 3, while Classes 1 and 2 showed similar, favorable survival. Cox proportional hazards regression analyses (Table 2) revealed that unadjusted trajectory patterns were associated with 28-day mortality (Class 1 as reference): Class 2 (HR 1.14, 95% CI 0.68–1.90, *p* = 0.619); Class 3 (HR 4.10, 95% CI 2.49–6.75, *p* < 0.001); Class 4 (HR 12.29, 95% CI 7.20-20.96, *p* < 0.001). After adjusting for comprehensive covariates (Model IV), Classes 3 and 4 remained independently associated with 28-day mortality risk: Class 3 (HR 2.79, 95% CI 1.55-5.00, *p* < 0.001); Class 4 (HR 4.76, 95% CI 2.33–9.72, *p* < 0.001). Similar associations were observed for 90-day mortality, with adjusted HRs of 1.21 (95% CI 0.83–1.78, *p* = 0.323), 2.34 (95% CI 1.49–3.68, *p* < 0.001), and 4.12 (95% CI 2.31–7.36, *p* < 0.001) for Classes 2, 3, and 4, respectively, compared to Class 1.

**Table 2:** Association between anion gap trajectories and 28-day and 90-day mortality.

AG trajectory	Model I		Model II		Model III		Model IV	
	HR (95% CI)	*P*	HR (95% CI)	*P*	HR (95% CI)	*P*	HR (95% CI)	*P*
28-day-mortality								
Class 1	1 (Ref)		1 (Ref)		1 (Ref)		1 (Ref)	
Class 2	1.14 (0.68–1.90)	0.619	1.12 (0.67–1.87)	0.658	1.34 (0.80–2.25)	0.265	1.22 (0.71–2.08)	0.471
Class 3	4.10 (2.49–6.75)	< 0.001	3.96 (2.40–6.54)	< 0.001	4.41(2.60–7.49)	< 0.001	2.79 (1.55-5.00)	0.001
Class 4	12.29 (7.20-20.96)	< 0.001	12.25 (7.18–20.91)	< 0.001	12.16 (6.89–21.45)	< 0.001	4.76 (2.33–9.72)	< 0.001
90-day-mortality								
Class 1	1 (Ref)		1 (Ref)		1 (Ref)		1 (Ref)	
Class 2	1.00 (0.70–1.43)	0.982	0.97 (0.68–1.39)	0.875	1.21 (0.84–1.73)	0.309	1.21 (0.83–1.78)	0.323
Class 3	2.66 (1.83–3.86)	< 0.001	2.55 (1.75–3.71)	< 0.001	3.27 (2.18–4.89)	< 0.001	2.34 (1.49–3.68)	< 0.001
Class 4	7.59 (4.97–11.6)	< 0.001	7.65 (5.01–11.7)	< 0.001	8.88 (5.63–14.01)	< 0.001	4.12 (2.31–7.36)	< 0.001

Model I: no adjusted.

Model II: adjusted for age, race, sex.

Model III: adjusted for Model II + etiology of acute cholangitis, AKI, renal disease, procedures of biliary drainage, hypertension, diabetes, sepsis.

Model IV: adjusted for Model III +bilirubin, hemoglobin, WBC, ALT, AST, CCI, SAPSII, SOFA.

AKI, acute kidney injury; WBC, white blood cell; ALT, alanine aminotransferase; AST, aspartate aminotransferase; CCI, Charlson comorbidity index; SAPS II, Simplified Acute Physiology Score II; SOFA, Sequential Organ Failure Assessment.

### Subgroup analyses

We conducted stratified analyses to evaluate associations between AG trajectories and 28- and 90-day mortality across predefined subgroups: age, sex, hypertension, and diabetes (Table 3). The trajectory-mortality association remained consistent across most subgroups. Notably, for 28-day mortality, interactions were observed with age (*p* for interaction = 0.013). In contrast, for 90-day mortality, a interaction was detected only with diabetes (*p* for interaction = 0.026).

**Table 3:** The relationship between serum anion gap trajectories and mortality in patients with AC in different subgroups.

Characteristics	Class 1	Class 2	Class 3	Class 4	*P* for interaction
28-day-mortality					
Age					0.013
≥ 65	1 (Ref)	0.81 (0.42–1.56)	2.28 (1.11–4.69)	4.45 (1.92–10.32)	
< 65	1 (Ref)	4.21 (1.49–11.94)	4.78 (1.45–15.71)	5.45 (1.26–23.5)	
Sex					0.089
Male	1 (Ref)	1.89 (0.91–3.92)	2.05 (0.86–4.86)	3.99 (1.47–10.84)	
Female	1 (Ref)	0.67 (0.28–1.64)	2.93 (1.24–6.91)	8.02 (2.51–25.65)	
Hypertension					0.326
Yes	1 (Ref)	1.21 (0.54–2.7)	1.69 (0.71–4.01)	3.41 (1.13–10.23)	
No	1 (Ref)	1.38 (0.65–2.93)	5.34 (2.36–12.08)	7.85 (2.86–21.50)	
Diabetes					0.606
Yes	1 (Ref)	1.26 (0.54–2.95)	1.83 (0.65–5.11)	4.47 (1.31–15.22)	
No	1 (Ref)	1.27 (0.61–2.61)	3.10 (1.46–6.62)	5.41 (2.01–14.55)	
90-day-mortality					
Age					0.053
≥ 65	1 (Ref)	0.98 (0.62–1.56)	1.91 (1.09–3.35)	4.05 (2.03–8.07)	
< 65	1 (Ref)	2.20 (1.03–4.66)	3.18 (1.30–7.80)	4.27 (1.26–14.4)	
Sex					0.074
Male	1 (Ref)	1.18 (0.71–1.98)	1.54 (0.81–2.92)	2.52 (1.13–5.63)	
Female	1 (Ref)	1.42 (0.78–2.60)	3.38 (1.70–6.73)	9.99 (3.94–25.31)	
Hypertension					0.296
Yes	1 (Ref)	0.93 (0.50–1.74)	1.16 (0.58–2.32)	1.81 (0.71–4.62)	
No	1 (Ref)	1.58 (0.95–2.62)	4.80 (2.60–8.89)	9.02 (4.06–20.04)	
Diabetes					0.026
Yes	1 (Ref)	0.64 (0.35–1.18)	0.98 (0.45–2.12)	2.25 (0.97–6.66)	
No	1 (Ref)	2.18 (1.26–3.77)	4.18 (2.25–7.76)	7.68 (3.34–17.66)	

Adjusted for age, race, sex, etiology of acute cholangitis, AKI, renal disease, procedures of biliary drainage, hypertension, diabetes, sepsis, bilirubin, hemoglobin, WBC, ALT, AST, CCI, SAPSII, SOFA.

AKI, acute kidney injury; WBC, white blood cell; ALT, alanine aminotransferase; AST, aspartate aminotransferase; CCI, Charlson comorbidity index; SAPS II, Simplified Acute Physiology Score II; SOFA, Sequential Organ Failure Assessment.

### Sensitivity analyses

To validate the robustness of the primary findings, sensitivity analyses were conducted excluding patients with ICU length of stay (LOS) < 1 day. In the restricted cohort (LOS ≥ 1 day), LCGM confirmed the four-class AG trajectory model as optimal (Supplementary Table S3 and Supplementary Figure S1). After adjustment for all covariates in multivariable Cox regression models, Classes 3 and 4 remained associated with elevated mortality risk: compared to Class 1, Class 3 exhibited a 28-day mortality HR of 3.14 (95% CI 1.63–6.05, *p* = 0.001) and 90-day HR of 2.94 (95% CI 1.75–4.94, *p* < 0.001), Class 4 demonstrated higher risk with 28-day HR 4.86 (95% CI 2.20–10.70, *p* < 0.001) and 90-day HR 4.70 (95% CI 2.46–8.97, *p* < 0.001). These results (Supplementary Table S4) further corroborate the robust association between adverse AG trajectories and increased mortality.

### Incremental predictive value of AG trajectories

We calculated the integrated discrimination improvement (IDI), and net reclassification improvement (NRI) based on Cox proportional hazards models (Table 4). At 28-day follow-up, the model incorporating both baseline AG and AG trajectory demonstrated significant improvements across all metrics compared with the baseline AG-only model: IDI 0.081 (95% CI 0.042–0.132; *p* < 0.001), NRI 0.231 (95% CI 0.119–0.370; *p* < 0.001). Similar improvements were observed at 90 days: IDI 0.043 (95% CI 0.015–0.076; *p* < 0.001). Collectively, AG trajectory confer significant incremental prognostic value beyond single baseline AG assessment, with consistent benefits at both 28-day and 90-day follow-up.

**Table 4:** Comparison of predictive performance between baseline AG and AG trajectory models.

Indicator	Baseline AG	Baseline AG + AG trajectory	*P*-value
28-days			
IDI	1 (Ref)	0.081 (0.042–0.132)	< 0.001
NRI	1 (Ref)	0.231 (0.119–0.370)	< 0.001
90-days			
IDI	1 (Ref)	0.043 (0.015–0.076)	< 0.001
NRI	1 (Ref)	0.118 (0.000-0.196)	0.052

IDI, Integrated Discrimination Improvement; NRI, Net Reclassification Improvement.

## Discussion

This retrospective cohort study utilized the MIMIC-IV database to investigate the association between AG trajectories and short- and long-term outcomes in patients with AC. Using LCGM to analyze AG values within the first 96 h after ICU admission, we identified four distinct trajectory groups: Class 1 with persistently low AG, Class 2 with sustained moderate levels, Class 3 with continuously high levels, and Class 4 characterized by a post-treatment decline but persistently elevated AG relative to other groups. Cox regression analysis identified Class 4 as the highest-risk stratum, with fully adjusted HRs of 4.76 (95% CI 2.33–9.72) for 28-day mortality and 4.12 (95% CI 2.31–7.36) for 90-day mortality compared to Class 1. These findings underscore the importance of dynamic AG monitoring for risk stratification in critically ill AC patients, suggesting that clinicians should maintain high vigilance for patients with persistently elevated AG and promptly intervene to correct acidosis and improve tissue perfusion.

As a biliary emergency, AC severity assessment primarily relies on Tokyo Guidelines 2018 (TG18). However, its six-domain evaluation system, encompassing cardiovascular, neurological, respiratory, coagulation, renal, and hepatic parameters, requires integrating 12 to 16 variables, rendering it impractical for rapid implementation in time-sensitive emergency and ICU settings^24^. The traditional Charcot’s triad has limited sensitivity for identifying critically ill patients due to suboptimal diagnostic performance^25,26^. While contemporary research has proposed several novel prognostic indicators, including CRP, PCT, lymphocyte count, neutrophil-to-lymphocyte ratio (NLR), AST-to-ALT ratio, systemic immune-inflammation index (SII), lipocalin-2, and sofa score^7,27–32^, these cross-sectional biomarkers cannot capture the temporal heterogeneity and dynamic progression of metabolic derangements in critically ill patients. We employed LCGM to identify distinct AG trajectory patterns, grouping patients with similar metabolic evolution to delineate associations between metabolic disturbance profiles and clinical outcomes. To our knowledge, this represents the first application of LCGM for mortality risk stratification in AC, providing a dynamic framework for prognostication.

As a biomarker of metabolic acidosis, the prognostic value of AG is being reassessed in critical care settings. Ji et al. established a positive correlation between AG elevation and all-cause mortality in adults^33^, while Li et al. confirmed AG > 16 mmol/L at ICU admission as an independent predictor of adverse outcomes in critically ill patients^14^. Huang et al. further established AG ≥ 18.13 mmol/L (equivalent to 18.13mEq/L) as a prognostic threshold for AC patients^18^. In our cohort, Class 4 patients maintained AG levels > 18 mmol/L despite therapeutic intervention, indicating sustained metabolic derangement as the primary mortality risk driver. This temporally dynamic risk profile, inherent in AG trajectory evolution, cannot be detected by conventional static prognostic models. The application of LCGM enables sophisticated risk stratification through intercept-slope parameterisation of individual AG trajectories, thereby establishing a clinically actionable framework for identifying high-risk AC subpopulations requiring precision interventions.

The AG is defined as serum sodium concentration minus the sum of chloride and bicarbonate concentrations (reference range: 8–16 mmol/L)^34,35^, quantifying the accumulation of unmeasured anions including lactate, ketone bodies, and phosphates. It serves as a key indicator of metabolic derangement severity in AC. Multiple pathophysiological mechanisms elucidate this association with adverse outcomes. Sustained AG elevation directly reflects persistent tissue hypoperfusion and anaerobic glycolysis^10,36^. Microcirculatory dysfunction induces hypoxia in hepatic and intestinal epithelia, activating the HIF-1α pathway to upregulate glycolysis and increase lactate production^37^. Concurrently, cholestasis-induced bile salt reflux impairs hepatocyte mitochondrial electron transport chains and suppresses pyruvate dehydrogenase complex activity, obstructing lactate oxidation^38–40^. Furthermore, AKI compromises renal compensatory mechanisms through diminished tubular NH₄⁺ excretion and H⁺ clearance, coupled with retention of fixed acids such as phosphates and sulfates, driving progressive AG elevation^41^. Critically, elevated AG exerts direct cytotoxicity: persistent accumulation of unmeasured anions—particularly lactate—attenuates myocardial contractility and impairs vascular responsiveness to catecholamines^42,43^, while activating NADPH oxidase-mediated reactive oxygen species (ROS) generation that exacerbates systemic inflammatory response syndrome (SIRS) and precipitates multiple organ dysfunction syndrome (MODS)^44–46^. This mechanistic cascade fully explains AG trajectory prognostic significance. Importantly, AG trajectory analysis initiated at ICU admission enables early identification of Class 4 patients through serial monitoring, providing a critical therapeutic window for interventions such as urgent endoscopic biliary drainage or renal replacement therapy during the golden period.

## Limitations of the study

This study has several inherent limitations. First, its retrospective design is susceptible to selection and information biases, and using a single-center database (MIMIC-IV) limits generalizability. Second, our analysis focused only on AG trajectories within the initial 96 h of ICU admission and did not evaluate longer-term temporal patterns. Finally, the observational nature of this study precludes definitive causal inference between AG dynamics and mortality. Consequently, our trajectory classifications, while clinically informative, warrant rigorous validation through prospective multicenter investigations.

## Conclusion

This study identified four heterogeneous AG trajectories in AC patients using LCGM. Among these, the trajectory characterized by a significantly elevated baseline AG that remained persistently > 18 mmol/L despite treatment exhibited the highest mortality risk, with 28-day and 90-day mortality risks 4.76-fold and 4.12-fold higher than the lowest-risk group, respectively. These findings suggest that dynamic AG trajectories serve as a precise prognostic stratification tool in AC, providing an objective basis for early identification of high-risk patients and implementation of targeted interventions.

## Supplementary Information

Below is the link to the electronic supplementary material.

Supplementary Material 1

## Data Availability

The datasets utilized for the analysis in this study can be accessed from the MIMIC-IV repository with the official link available at https:/mimic.mit.edu.

## Abbreviations

AG: Anion gap
AC: Acute cholangitis
MIMIC-IV: Medical Information Mart for Intensive Care IV
LCGM: Latent class growth modeling
CRP: C-reactive protein
ICU: Intensive care unit
ICD: International Classification of Diseases
HR: Heart rate
RR: Respiratory rate
SpO_2_: Oxygen saturation
PLT: Platelet count
WBC: White blood cell
BUN: Blood urea nitrogen
INR: International normalized ratio
ALT: Alanine aminotransferase
AST: Aspartate aminotransferase
AKI: acute kidney injury
CRRT: Continuous renal replacement therapy
CHF: Congestive heart failure
COPD: Chronic obstructive pulmonary disease
CCI: Charlson Comorbidity Index
SAPS II: Simplified Acute Physiology Score II
SOFA: Sequential Organ Failure Assessment
HR: Hazard ratios
CI: Confidence intervals
AIC: Akaike Information Criterion
BIC: Bayesian Information Criterion
SABIC: sample size-adjusted BIC
NLR: neutrophil-to-lymphocyte ratio
IDI: Integrated Discrimination Improvement
NRI: Net Reclassification Improvement
