# Supplementary material for: Association between anion gap trajectories and mortality in patients with acute cholangitis: a cohort study
Source: Sci Rep. 2026 Apr 10;16:16446. doi: 10.1038/s41598-026-48549-5 (PMC13216307; doi:10.1038/s41598-026-48549-5)
Supplement: Supplementary file 1 — Supplementary Material 1 [file 41598_2026_48549_MOESM1_ESM.docx]

# **Association between anion gap trajectories and mortality in patients with acute cholangitis: a cohort study**

[Yanhua Chen](https://pubmed.ncbi.nlm.nih.gov/?term=Chen+Y&cauthor_id=37749449)^1,2*^, Guirong Xiao^3^, Ling Mou^4^

^1^Department of Pharmacy, Hospital of Chengdu University of Traditional Chinese Medicine, Chengdu, China

^2^School of Clinical Medicine, Chengdu University of Traditional Chinese Medicine, Chengdu, China.

^3^Department of Pharmacy , West China Hospital, Sichuan University, Chengdu，China

^4^Department of Pharmacy, Traditional Chinese Medicine Hospital of Meishan, Meishan, China

*** Correspondence:**

[Yanhua Chen](https://pubmed.ncbi.nlm.nih.gov/?term=Chen+Y&cauthor_id=37749449)

chenyanhua@cdutcm.edu.cn

**Supplementary Table**

**Table S1.** The latent class growth modeling (LCGM) model parameters for anion gap level trajectory grouping.

**Table S2.** Mean posterior probability under four classes of anion gap level trajectory.

**Table S3.** The latent class growth modeling (LCGM) model parameters for anion gap level trajectory grouping.

**Table S4.** The relationship between the anion gap levels and mortality in patients with acute cholangitis.**Supplementary Table S1**. The latent class growth modeling (LCGM) model parameters for anion gap level trajectory grouping.

| **Number of classes** | **Log likelihood** | **AIC** | **BIC** | **SABIC** | **Entropy** | **Class 1 (%)** | **Class 2 (%)** | **Class 3 (%)** | **Class 4 (%)** | **Class 5 (%)** |
| --- | --- | --- | --- | --- | --- | --- | --- | --- | --- | --- |
| 2 | -12677.09 | 25384.18 | 25455.55 | 25407.91 | 0.65 | 85.13 | 14.87 |  |  |  |
| 3 | -12653.40 | 25346.80 | 25441.96 | 25378.45 | 0.56 | 63.65 | 29.04 | 7.31 |  |  |
| 4 | -12636.17 | 25322.34 | 25441.29 | 25361.90 | 0.61 | 29.97 | 46.81 | 17.18 | 6.04 |  |
| 5 | -12625.54 | 25311.08 | 25453.82 | 25358.55 | 0.66 | 33.34 | 38.56 | 6.85 | 5.92 | 15.33 |

Abbreviations: AIC, Akaike Information Criterion; BIC, Bayesian Information Criterion; SABIC, sample-size-adjusted Bayesian Information Criterion. **Supplementary Table S2**. Mean posterior probability under four classes of anion gap level trajectory.

|  | **Class 1** | **Class 2** | **Class 3** | **Class 4** |
| --- | --- | --- | --- | --- |
| Probability | 0.7950 | 0.7440 | 0.7937 | 0.8834 |

**Supplementary Table S3**. The latent class growth modeling (LCGM) model parameters for anion gap level trajectory grouping.

| **Number of classes** | **Log likelihood** | **AIC** | **BIC** | **SABIC** | **Entropy** | **Class 1 (%)** | **Class 2 (%)** | **Class 3 (%)** | **Class 4 (%)** | **Class 5 (%)** |
| --- | --- | --- | --- | --- | --- | --- | --- | --- | --- | --- |
| 2 | -11181.86 | 22393.73 | 22462.63 | 22415.00 | 0.63 | 83.15 | 16.85 |  |  |  |
| 3 | -11159.63 | 22359.26 | 22451.12 | 22387.61 | 0.68 | 76.71 | 1.78 | 21.51 |  |  |
| 4 | -11142.18 | 22334.37 | 22449.19 | 22369.81 | 0.62 | 29.04 | 47.40 | 17.26 | 6.30 |  |
| 5 | -11134.58 | 22329.15 | 22466.94 | 22371.68 | 0.62 | 11.10 | 20.41 | 41.64 | 7.12 | 19.73 |

Abbreviations: AIC, Akaike Information Criterion; BIC, Bayesian Information Criteria; SABIC, sample-size-adjusted Bayesian Information Criterion. The data has excluded patients who stayed in the ICU for less than one day.**Supplementary Table S4**. The relationship between the anion gap levels and mortality in patients with acute cholangitis.

| **AG trajectory** | **Model I** | | **Model II** | | **Model Ⅲ** | | | | | **Model Ⅳ** | |
| --- | --- | --- | --- | --- | --- | --- | --- | --- | --- | --- | --- |
|  | **HR (95%CI)** | ***P*** | **HR (95%CI)** | ***P*** | **HR (95%CI)** | | | ***P*** | | **HR (95%CI)** | ***P*** |
| **28-day-mortality** | | | | | | | | | | | |
| Class 1 | 1(Ref) |  | 1(Ref) |  | | 1(Ref) |  | | 1(Ref) | |  |
| Class 2 | 1.52 (0.87-2.67) | 0.145 | 1.48 (0.84-2.60) | 0.174 | | 1.77 (1.00-3.14) | 0.05 | | 1.57 (0.87-2.81) | | 0.132 |
| Class 3 | 4.86 (2.77-8.54) | < 0.001 | 4.60 (2.61-8.10) | < 0.001 | | 4.88 (2.68-8.9) | < 0.001 | | 3.14 (1.63-6.05) | | 0.001 |
| Class 4 | 12.16 (6.65-22.26) | < 0.001 | 12.15 (6.64-22.24) | < 0.001 | | 11.72 (6.16-22.29) | < 0.001 | | 4.86 (2.20-10.7) | | < 0.001 |
| **90-day-mortality** | | | | | | | | | | | |
| Class 1 | 1(Ref) |  | 1(Ref) |  | | 1(Ref) |  | | 1(Ref) | |  |
| Class 2 | 1.33 (0.89-1.97) | 0.163 | 1.27 (0.85-1.89) | 0.241 | | 1.60 (1.07-2.41) | 0.022 | | 1.67 (1.08-2.56) | | 0.020 |
| Class 3 | 3.24 (2.13-4.93) | < 0.001 | 3.09 (2.03-4.73) | < 0.001 | | 3.93 (2.49-6.20) | < 0.001 | | 2.94 (1.75-4.94) | | < 0.001 |
| Class 4 | 7.95 (4.95-12.77) | < 0.001 | 8.01 (4.98-12.88) | < 0.001 | | 9.27 (5.56-15.45) | < 0.001 | | 4.70 (2.46-8.97) | | < 0.001 |

The data has excluded patients who stayed in the ICU for less than one day. Model I: no adjusted; Model II: adjusted for age, race, sex; Model Ⅲ: adjusted for Model II + etiology of acute cholangitis, AKI, renal disease, procedures of biliary drainage, hypertension, diabetes, sepsis; Model Ⅳ: adjusted for Model Ⅲ +bilirubin, hemoglobin, WBC, ALT, AST, CCI, SAPSII, SOFA

Abbreviations: AKI, acute kidney injury; WBC, white blood cell count; ALT, alanine aminotransferase; AST, aspartate aminotransferase; CCI, Charlson comorbidity index; SAPS II, Simplified Acute Physiology Score II; SOFA, Sequential Organ Failure Assessment. **Supplementary Figure 1**

**
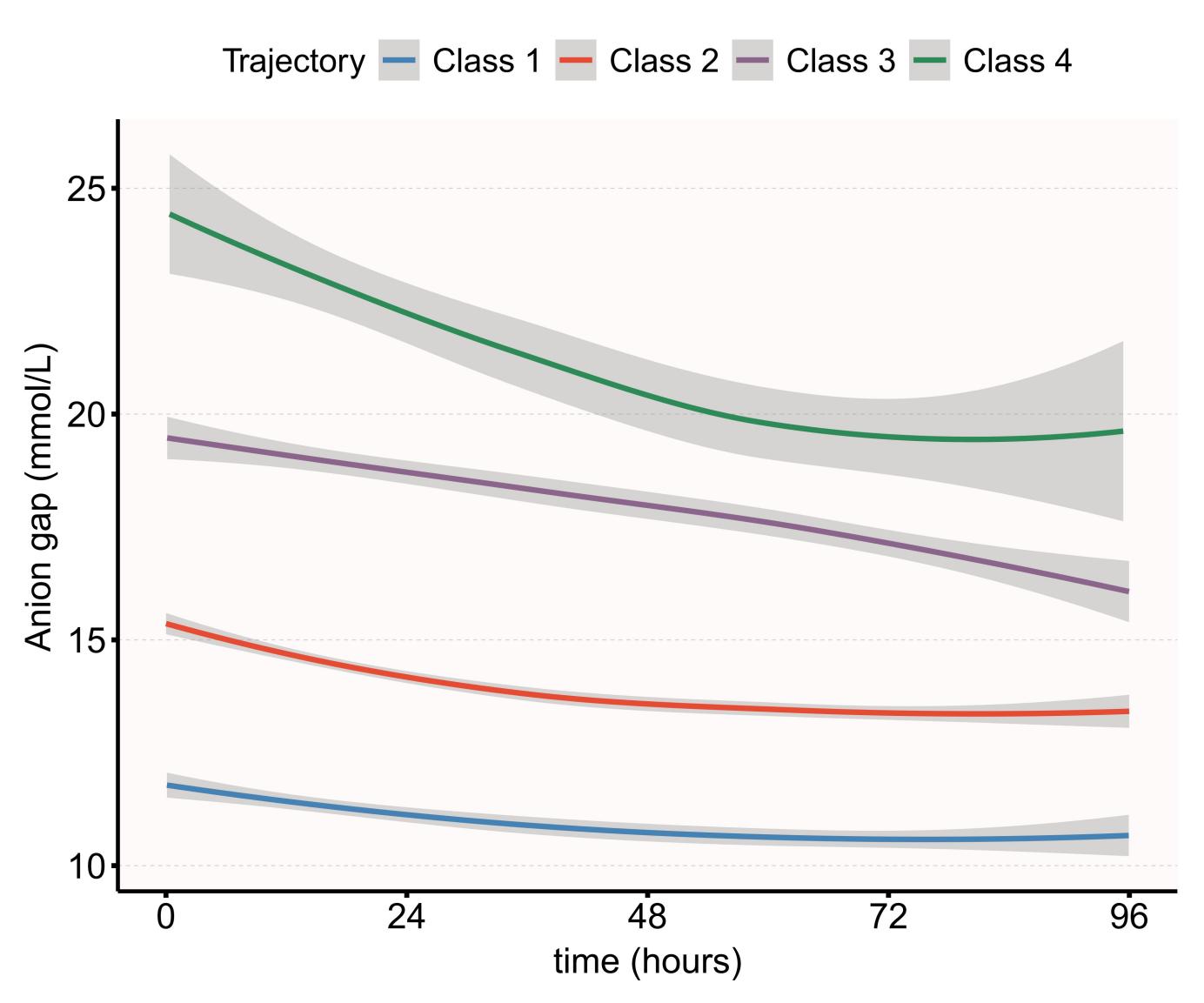
**

**Supplementary Figure 1.** AG trajectory during the first 96 hours after ICU admission. The data has excluded patients who stayed in the ICU for less than one day.
